# Insights into the single cell draft genome of “*Candidatus* Achromatium palustre”

**DOI:** 10.1186/s40793-016-0146-x

**Published:** 2016-03-23

**Authors:** Verena Salman, Tom Berben, Robert M. Bowers, Tanja Woyke, Andreas Teske, Esther R. Angert

**Affiliations:** Cornell University, Ithaca, NY USA; University of Amsterdam, Amsterdam, The Netherlands; DOE Joint Genome Institute, Walnut Creek, CA USA; University of North Carolina, Chapel Hill, NC USA

**Keywords:** “*Candidatus* Achromatium palustre”, Large sulfide-oxidizing bacteria, *Thiotrichaceae*, Calcium carbonate, Sippewissett Salt Marsh

## Abstract

“*Candidatus* Achromatium palustre” was recently described as the first marine representative of the *Achromatium* spp. in the *Thiotrichaceae* - a sister lineage to the *Chromatiaceae* in the *Gammaproteobacteria. Achromatium* spp. belong to the group of large sulfur bacteria as they can grow to nearly 100 μm in size and store elemental sulfur (S^0^) intracellularly. As a unique feature, *Achromatium* spp. can accumulate colloidal calcite (CaCO_3_) inclusions in great amounts. Currently, both process and function of calcite accumulation in bacteria is unknown, and all *Achromatium* spp. are uncultured. Recently, three single-cell draft genomes of *Achromatium* spp. from a brackish mineral spring were published, and here we present the first draft genome of a single “*Candidatus* Achromatium palustre” cell collected in the sediments of the Sippewissett Salt Marsh, Cape Cod, MA. Our draft dataset consists of 3.6 Mbp, has a G + C content of 38.1 % and is nearly complete (83 %). The next closest relative to the *Achromatium* spp. genomes is *Thiorhodovibrio* sp. 907 of the family *Chromatiaceae*, containing phototrophic sulfide-oxidizing bacteria.

## Introduction

*Achromatium* spp. have been known for over a century and have been detected in sediments of freshwater [1–5] and marine [6, 7] environments. They are large rod-shaped bacteria that typically range in size from 5–40 μm in diameter and 15–100 μm in length, and they migrate by slow rolling along the opposing sedimentary redox gradients of sulfide and oxygen [8]. The first species described was *Achromatium oxaliferum*, named after the large intracellular inclusions, which were suggested to consist of calcium oxalate [5]. Later it was found that they are actually composed of calcium carbonate, also referred to as calcite [1, 3, 9]. To this day, *Achromatium* spp. remain uncultured and their ecophysiology has been investigated in freshwater populations, mainly using microcosm experiments [2, 8, 10–13]. *Achromatium* spp. are presumably chemolithotrophic, and oxidize reduced sulfur compounds completely to sulfate [11, 13, 14], they are suggested to be microaerophilic, and may use nitrate as alternative electron acceptor to oxygen [3, 10, 13–16].

A marine population of *Achromatium* spp. [6] was recently described in more detail [7] and this population showed altered migration patterns as well as an increased tolerance to oxygen as reported for freshwater populations [14]. Besides calcite and sulfur inclusions, staining and energy dispersive X-ray analysis revealed a third type of inclusion in the salt marsh *Achromatium* containing a high concentration of Ca^2+^ ions that were suggested to be stored for the rapid, dynamic precipitation of calcium carbonate. The number of inclusions varied according to the position of a cell relative to the redox gradient of the sediment [7].

Sequencing *Achromatium* genomes not only provides insight into the genetic and ecophysiological potential of these uncultured organisms in order to find genetic evidence supporting field and microcosm observations (Table 1), but also enables the identification of candidate genes involved in calcite accumulation. Three draft genomes of *Achromatium* from a mineral spring in Florida were recently published [17], and here we present the first draft genome of a marine *Achromatium* representative.

**Table 1:** Classification and general features of “*Candidatus* Achromatium plaustre” according to the MIGS recommendations [40]

MIGS ID	Property	Term	Evidence code^a^
	Classification	Domain *Bacteria*	TAS [41]
		Phylum *Proteobacteria*	TAS [42–44]
		Class *Gammaproteobacteria*	TAS [44, 45]
		Order *Thiotrichales*	TAS [32]
		Family *Thiotrichaceae*	TAS [31]
		Genus *Achromatium*	TAS [5, 46]
		Species *Candidatus* Achromatium palustre	TAS [7, 47]
	Gram stain	Negative	TAS [14]
	Cell shape	Rod/coccus/variable	TAS [7]
	Motility	Motile	TAS [7]
	Sporulation	Not reported	NAS
	Temperature range	Candidatus 10–30 °C	TAS [7]
	Optimum temperature	Not reported	NAS
	pH range	Candidatus 5–9	TAS [7]
	Carbon source	Autotroph, heterotroph	TAS [11]
MIGS-6	Habitat	Aquatic, marine sediment	TAS [7]
MIGS-6.3	Salinity	Candidatus 3.5 % NaCl (w/v)	TAS [7]
MIGS-22	Oxygen requirement	Aerobic/microaerophilic/aerotolerant	TAS [7]
MIGS-15	Biotic relationship	Free-living	TAS [7]
MIGS-14	Pathogenicity	Non-pathogenic	NAS
MIGS-4	Geographic location	Cape Cod, MA, Sippewissett Salt Marsh	TAS [7]
MIGS-5	Sample collection	August 2014	TAS [7]
MIGS-4.1	Latitude	41.575804	TAS [7]
MIGS-4.2	Longitude	−70.639768	TAS [7]
MIGS-4.4	Altitude	0 m	TAS [7]

^a^TAS: Traceable Author Statement (i.e., a direct report exists in the literature); NAS: Non-traceable Author Statement (i.e., not directly observed for the living, isolated sample, but based on a generally accepted property for the species, or anecdotal evidence). These evidence codes are from the Gene Ontology project [48]

## Organism information

### Classification and features

**Fig. 1:**
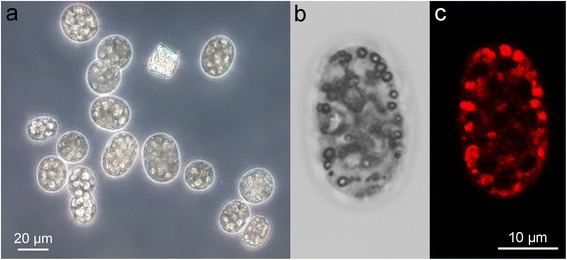
Micrographs of *Candidatus* Achromatium palustre. **a** Light micrograph showing that each cell contains large bulgy calcite inclusions, which highly reflect the light. The square-shaped, reflective organism in the *top middle* is a diatom. **b** and **c** show the same cell imaged with a confocal microscope; **b** is taken with transmitted light showing smaller inclusions between the large calcite inclusions, and **c** is the fluorescent signal of Calcium Orange-5 N showing the co-localization of highly concentrated Ca^2+^ ions (*bright red*) with the smaller granules visible in (**b**)

As the most striking phenotypic feature, *Candidatus* A. palustre, as well as other described *Achromatium* species, appear bright white to the naked eye, as they contain multiple intracellular calcium carbonate (CaCO_3_) inclusions, and elemental sulfur (S^0^) granules, that fill nearly the entire interior of the cell. There is no large central vacuole as observed in other large sulfur bacteria, e.g. *Beggiatoa* spp. [18]. Calcite inclusions vary in diameter, but are typically several micrometers in size. Under the microscope, *Achromatium* spp. appear bulgy and rock-like (Fig. 1a), and one can observe the slowly jerky rolling motility of the large cells. TEM imaging of freshwater *Achromatium* showed that the calcite inclusions have a central nucleation point that is surrounded by concentric rings of precipitated calcite, and that they are probably enclosed by a membrane [14]. The salt marsh *Achromatium* were on average 20 × 26 μm in diameter, rod-shaped, contained several large calcite inclusions, and numerous small interstitial inclusions. Some cells had an external sheath, supposedly a layer of mucus, to which occasionally other rod-shaped and filamentous bacteria were attached [7]. Staining with Calcium Orange-5 N (Fig. 1c), or Calcium Green-1 revealed additional inclusions that were highly enriched in Ca^2+^ and of much smaller size (<1 μm) in the interstitial space between the large calcite inclusions (compare Fig. 1b and c) [7]. *Achromatium* have a Gram-negative cell wall [3, 19], and the cytoplasm as well as DNA is distributed across the entire cell in thin (<2 μm) threads stretching between the inclusions [7].

*Candidatus* Achromatium palustre was detected in Little Sippewissett Salt Marsh on Cape Cod, Massachusetts, where they occurred mainly in the upper 2 cm of the sediment of a tide pool. From the deeper layers of the flocculous, organic-rich phytodetritus, high sulfide concentrations diffused upwards meeting the sediment/water interface during the night. During the day, photosynthetic algae and cyanobacteria generated supersaturated oxygen concentrations in the surficial sediment and overlying water column, which created an oxic, sulfide-free zone in the upper millimeters of the sediment [7].

The salt marsh *Achromatium* population co-occurred with highly abundant and conspicuous, millimeter-size aggregates of purple sulfur bacteria in the surficial sediment layers. Other phototrophic bacteria (phylum *Cyanobacteria*) and eukaryotes (diatoms) are also found in higher densities at the sediment/water interface; heterotrophic sulfate-reducing bacteria of the *Deltaproteobacteria* dominate in deeper sediment layers [7, 20, 21]. The single *Candidatus* A. palustre cell was isolated by an initial sieving of the sediment to remove the large aggregates and debris, followed by manual removal of the cell using a glass Pasteur pipette, and a successive washing steps in sterile water until contaminants were out-diluted.

**Fig. 2:**
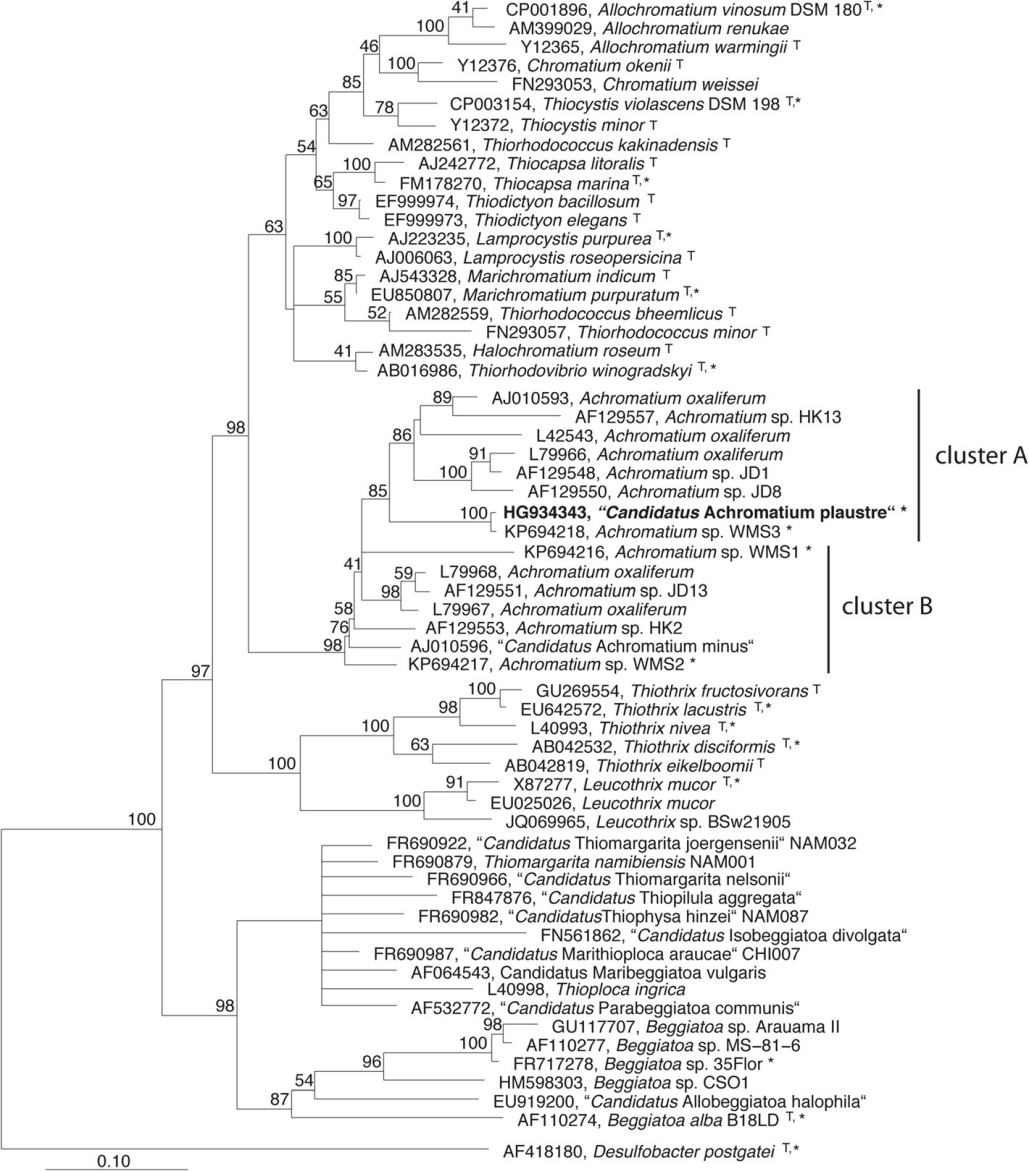
Phylogenetic tree based on 16S rRNA gene sequence information. The reconstruction was performed originally with 80 sequences, of which only a subset is shown here, and a total of aligned 1,101 positions using the maximum likelihood RaxML method of the ARB software package [49]. The tree was rooted with representatives of the *Deltaproteobacteria.* Branching patterns supported by <40 % confidence in 100 bootstraps replicates were manually converted into multifurcations. *Candidatus* Achromatium palustre, the source organism of the here presented genome, affiliates with cluster A in the *Achromatium* lineage, and is highlighted in bold face. (T) marks type strains/sequences, and asterisks (*) shows the availability of a genome

Currently, *Achromatium* spp. 16S rRNA gene sequences are either classified as *Achromatium oxaliferum*, or *Achromatium* sp., intermixed [2, 3, 22] between the two phylogenetic subclusters “A” and “B” (Fig. 2). These subclusters not only separate by 16S rRNA gene sequence difference, but also by the presence (A) or absence (B) of helix 38 in the V6 region [2]. Recently, it was proposed that the subclusters may represent and/or include several *candidatus* taxa [8], however, due to the lack of cultures, a reclassification of the members of the *Achromatium* lineage is challenging, as it cannot be based on sequence information alone [23]. With the accumulation of information about the natural populations and subpopulations through culture-independent techniques the phylotypes will most likely receive phylogenetic attention in the future. One subcluster in “cluster B” was already classified as “*Candidatus* Achromatium minus” based on sequence divergence and morphological difference [24]. “*Candidatus* Achromatium palustre” was likewise classified as part of “cluster A”, based on 16S rRNA gene sequence information and their adaptation to the very different habitat, as well as their altered behavioural characteristics [7] (Fig. 2).

*Achromatium* spp. have originally been classified in the family *Achromatiaceae* [25, 26] as a sister family of the *Beggiatoaceae* [27] and *Leucotrichaceae* [28] within the order *Beggiatoales* [29, 30]. Recently, a reclassification was published [31], merging these families into one newly created family *Thiotrichaceae* (Table 1), in the order *Thiotrichales* [32].

## Genome sequencing information

### Genome project history

The sequencing project was initiated in August 2013, when cells were collected from the field, isolated, and subjected to multiple displacement amplification. The amplified DNA was sequenced in November 2014, the raw data were integrated into the JGI pipeline Jigsaw2.4.1, where they were quality-checked and assembled. Annotation and further decontamination was performed through IMG [33]. After final analysis for contamination and completion in CheckM [34], the draft genome (Table 2) was completed in February 2015, when it was deposited in the Genome On-Line Database and became available in IMG (Ga0065144). The whole genome shotgun project has been deposited at DDBJ/EMBL/GenBank under the accession number LFCU00000000.

**Table 2:** Genome sequencing project information

MIGS ID	Property	Term
MIGS 31	Finishing quality	Draft
MIGS-28	Library used	TruSeq DNA PCR-Free Library Prep Kit
MIGS 29	Sequencing platform	Illumina MiSeq v2
MIGS 31.2	Fold coverage	375x
MIGS 30	Assembler	Spades 3.5.0
MIGS 32	Gene calling methods	IMG: tRNAScan-SE-1.23, BLAST search for rRNAs, CRT for CRISPRS, infernal and rfam_scan for other rRNAs, GeneMark for protein coding genes
	Locus Tag	AC002
	Genbank ID	3742159
	GenBank Date of Release	1 July, 2015
	GOLD ID	Ga0065144
	BIOPROJECT	PRJNA251325
MIGS 13	Source Material Identifier	Environmental sample
	Project relevance	Metabolic pathways, non-pathogenic

### Growth conditions and genomic DNA preparation

The cell was retrieved directly from the field, added to the sample buffer of the illustra GenomiPhi V2 kit (GE Healthcare Life Sciences, Pittsburgh, PA), crushed manually with a sterile needle, heated for 3 min at 95 °C, and supplemented with the remaining ingredients for the MDA reaction [35]. Purity of the MDA product was assessed by amplifying the 16S rRNA gene sequence and directly sequencing the PCR product with Sanger. The genome was then reamplified with the illustra GenomiPhi HY DNA Amplification kit to yield enough material for whole genome sequencing.

### Genome sequencing and assembly

The MDA product was sequenced with illumina MiSeq v2 technology at the Cornell University Institute of Biotechnology, Ithaca, NY. This resulted a total of 30,190,768 reads, which were quality checked, trimmed, and artifact/contamination filtered with DUK, a filtering program developed at the JGI that removes known Illumina sequencing and library preparation artifacts. Additionally, reads were screened for human, cat, and dog contaminant sequences. The remaining 29,696,136 reads were passed to SPAdes [36] and assembled into 586 contigs >2 kb, representing 7,614,708 bp. This dataset was uploaded in IMG/mer [37] under analysis project number Ga0064002, and further decontaminated manually. Only contigs affiliating with the *Thiotrichaceae**/**Chromatiales* lineage were finally uploaded in IMG/er [38] under analysis project number Ga0065144. This final dataset is the draft genome of *Candidatus* A. palustre and consists of 3,645,683 bp on 276 contigs, and the coverage is 375x. CheckM is software that is designed to assess quality and completeness of (meta)genomes [34], and our analysis of the draft genome dataset revealed a completeness of 83.36 % based on the finding of 503/538 lineage specific maker genes (marker lineage *Gammaproteobacteria*), and a contamination value of 1.13 %, which is in the error range (≤6 %) of contamination estimates of incomplete (~70 %) genomes [34]. Strain heterogeneity, tested by the amino acid identity (AAI) between multi-copy genes [34], is 0.

### Genome annotation

Gene calling and functional annotation was performed automatically by IMG [33, 39] during the upload process. We are currently manually verifying annotations of interest, constructing databases using Uniprot (Swissprot and TrEMBL) and blasting against these with the *Achromatium* draft genome using the integrated tblastn tool in IMG/er.

## Genome properties

**Fig. 3:**
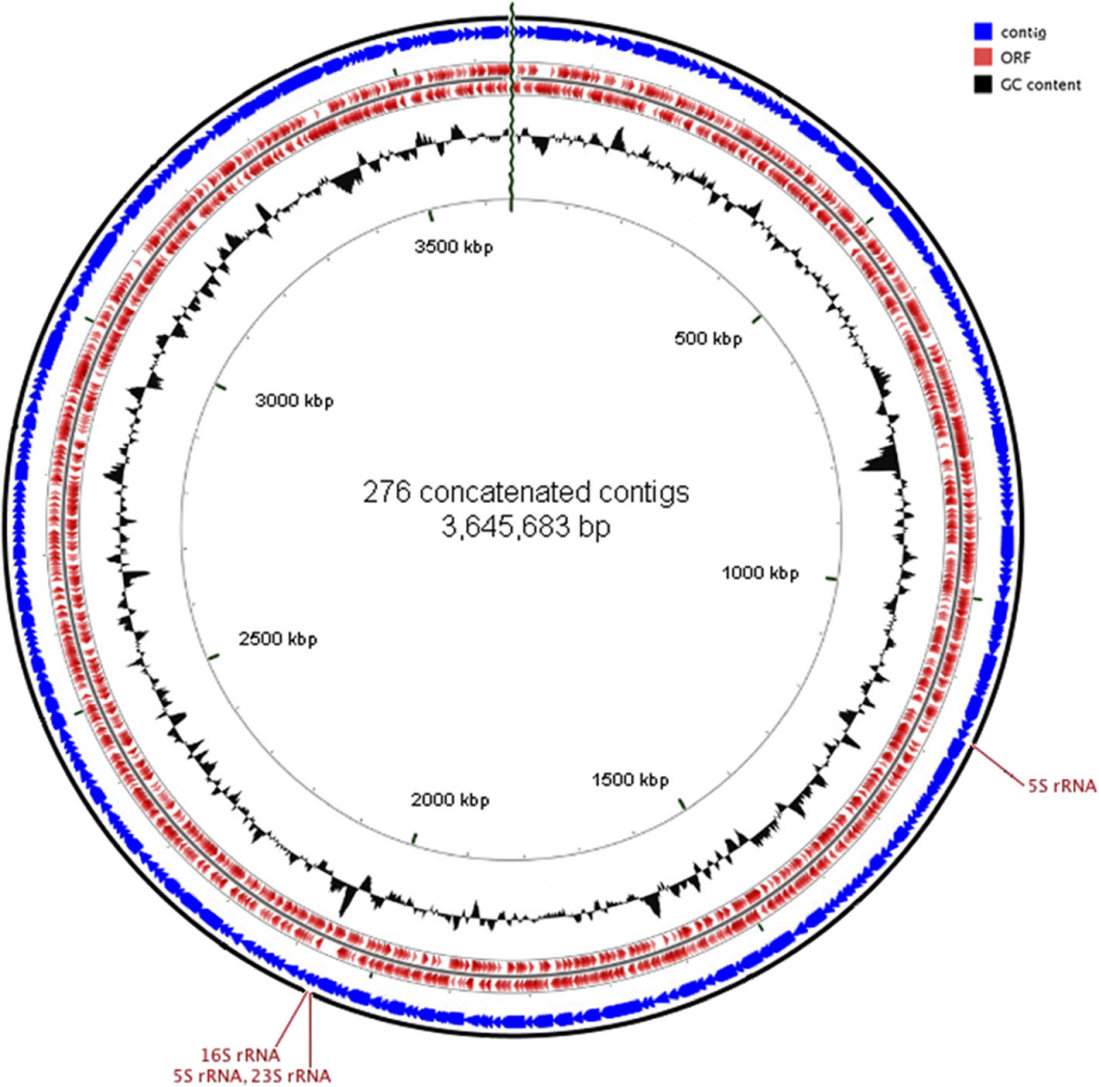
Graphical simulated circular genome of 276 concatenated contigs of the *Candidatus* A. palustre draft genome. The contigs were concatenated in Geneious 6.0.1 [50] using the random order of appearance in IMG, and the map was generated in Geneious and CGView [51]. The concatenated contigs are shown in *blue*, open reading frames (ORFs) in *red* in both directions, and the GC content in *black*

The *Candidatus* Achromatium palustre draft genome is 3,645,683 bp in size, and distributed on 276 contigs that are between 2012 and 57,118 bp in length. The N_50_ is 18,361 bp, and the G + C content is 38.08 %. Based on sequence comparison of nearly full-length 16S rRNA genes, the phylogenetic affiliation of the *Candidatus**Achromatium* palustre genome is in cluster A among other *Achromatium* spp. sequences, including the three previously published draft genomes (Fig. 2). The *Achromatium* lineage is a sister lineage to the *Chromatiaceae* [2, 3, 8, 22, 24] containing purple sulfur bacteria such as *Thiorhodovibrio* and *Chromatium* (Fig. 2). IMG identified 3,400 genes, of which 3,343 encoded proteins (98.32 %), 57 encoded rRNA (1.68 %) and no pseudogenes (0.00 %). Among the 57 rRNA genes, one operon contained the 16S rRNA, 23S rRNA, and 5S rRNA gene. An additional truncated 5S rRNA gene was located on a different contig, and the sequence is identical to the full-length 5S rRNA gene. Furthermore, we find, e.g., 42 tRNA genes, genes for transcription and translation, DNA replication and repair, cell motility and chemotaxis. Details are given in Fig. 3, and Tables 3 and 4. We did not identify indications for plasmid DNA.

**Table 3:** Genome statistics

Attribute	Value	% of total
Genome size (bp)	3,645,683	100.00
DNA coding (bp)	2,985,540	81.89
DNA G + C (bp)	1,388,144	38.08
DNA scaffolds	276	
Total genes	3,400	100.00
Protein coding genes	3,343	98.32
RNA genes	57	1.68
Pseudo genes	0	0.00
Genes in internal clusters	NA	
Genes with function prediction	2,259	66.44
Genes assigned to COGs	1,678	49.35
Genes with Pfam domains	2,343	68.91
Genes with signal peptides	187	5.50
Genes with transmembrane helices	799	23.50
CRISPR repeats	9

**Table 4:** Number of genes associated with general COG functional categories

Code	Value	% age	Description
J	167	5.00	Translation, ribosomal structure and biogenesis
A	1	0.03	RNA processing and modification
K	51	1.52	Transcription
L	67	2.00	Replication, recombination and repair
B	1	0.03	Chromatin structure and dynamics
D	25	0.75	Cell cycle control, Cell division, chromosome partitioning
V	81	2.42	Defense mechanisms
T	126	3.77	Signal transduction mechanisms
M	156	4.67	Cell wall/membrane biogenesis
N	53	1.59	Cell motility
U	28	0.84	Intracellular trafficking and secretion
O	135	4.04	Posttranslational modification, protein turnover, chaperones
C	128	3.83	Energy production and conversion
G	56	1.68	Carbohydrate transport and metabolism
E	131	3.92	Amino acid transport and metabolism
F	46	1.38	Nucleotide transport and metabolism
H	95	2.84	Coenzyme transport and metabolism
I	54	1.62	Lipid transport and metabolism
P	82	2.45	Inorganic ion transport and metabolism
Q	21	0.63	Secondary metabolites biosynthesis, transport and catabolism
R	191	5.71	General function prediction only
S	92	2.75	Function unknown
-	1722	51.51	Not in COGs

The total is based on the total number of protein coding genes in the genome

Further insights into the coding regions of the draft genome will be given elsewhere.

## Conclusions

Details of *Achromatium* spp. genomes promise further insight into the ecophysiology of these unique organisms. The draft genome of *Candidatus* A plaustre is one of the first steps to unravel the phenotypic and physiological adaptations of *Achromatium* spp. occurring in different redox gradient systems as well as across divers salinities. A comparison with the brackish *Achromatium* genomes and prospect freshwater *Achromatium* spp. genomes, as well as with future metagenomes of different *Achromatium*-containing habitats, will be conducted and promise highly valuable information. Future analyses will not only include the investigation of nutrient pathways and modes of energy generation in these organisms, but also potential insights into calcium transport and calcite accumulation.
